# Angiogenic Properties of NK Cells in Cancer and Other Angiogenesis-Dependent Diseases

**DOI:** 10.3390/cells10071621

**Published:** 2021-06-29

**Authors:** Dorota M. Radomska-Leśniewska, Agata Białoszewska, Paweł Kamiński

**Affiliations:** 1 Center for Biostructure Research, Department of Histology and Embryology, Medical University of Warsaw, 02-004 Warsaw, Poland; dradomska@wum.edu.pl; 2 Department of Gynecology and Gynecological Oncology, Military Institute of Medicine, 04-349 Warsaw, Poland; pawel.kaminscy@gmail.com

**Keywords:** NK cells, decidual NK cells, angiogenesis, proangiogenic factors, tumor microenvironment, endometriosis, rheumatoid arthritis, VEGF, TGF-β, hypoxia

## Abstract

The pathogenesis of many serious diseases, including cancer, is closely related to disturbances in the angiogenesis process. Angiogenesis is essential for the progression of tumor growth and metastasis. The tumor microenvironment (TME) has immunosuppressive properties, which contribute to tumor expansion and angiogenesis. Similarly, the uterine microenvironment (UME) exerts a tolerogenic (immunosuppressive) and proangiogenic effect on its cells, promoting implantation and development of the embryo and placenta. In the TME and UME natural killer (NK) cells, which otherwise are capable of killing target cells autonomously, enter a state of reduced cytotoxicity or anergy. Both TME and UME are rich with factors (e.g., TGF-β, glycodelin, hypoxia), which support a conversion of NK cells to the low/non-cytotoxic, proangiogenic CD56^bright^CD16^low^ phenotype. It is plausible that the phenomenon of acquiring proangiogenic and low cytotoxic features by NK cells is not only limited to cancer but is a common feature of different angiogenesis-dependent diseases (ADDs). In this review, we will discuss the role of NK cells in angiogenesis disturbances associated with cancer and other selected ADDs. Expanding the knowledge of the mechanisms responsible for angiogenesis and its disorders contributes to a better understanding of ADDs and may have therapeutic implications.

## 1. Introduction

Angiogenesis, a new blood vessel formation from preexisting vasculature, is a normal and vital process in growth and development. Physiologically, angiogenesis regulates embryogenesis, the menstrual cycle, wound healing, and the formation of granulation tissue [1].

Disturbances in neovascularization can lead to serious health consequences since the pathogenesis of many severe conditions is closely related to angiogenic disorders. These conditions are called angiogenesis-dependent diseases (ADDs) and are characterized by a disturbed formation of new vessels, which affects their organization, structure, and function [2]. ADDs comprise conditions with excessive pathological angiogenesis, such as cancer, some eye diseases (e.g., age-related macular degeneration [AMD]), or chronic inflammatory disorders (e.g., rheumatoid arthritis [RA], psoriasis) and diseases with insufficient angiogenesis (e.g., diabetes mellitus, stroke, atherosclerosis, hypertension, or ischemic heart disease) [2,3].

**Figure 1:**
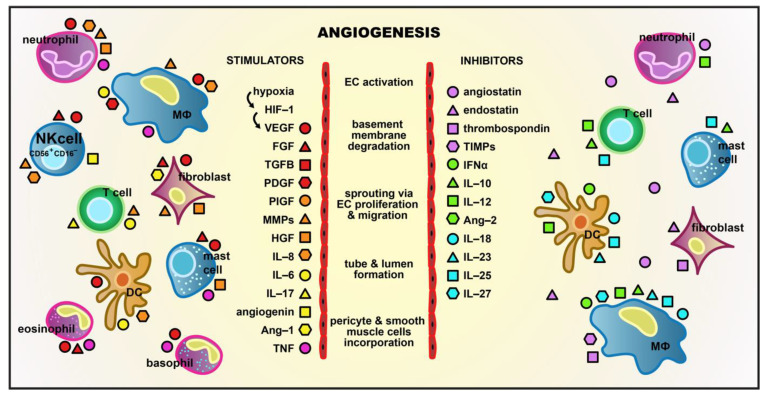
Stages of the angiogenesis process and its regulation. New blood vessel formation is upregulated by hypoxia, which stimulates hypoxia-inducible factor-1 (HIF-1) leading to the vascular endothelial growth factor (VEGF) expression. VEGF, fibroblast growth factor (FGF), transforming growth factor (TGF), platelet-derived growth factor (PDGF), hepatocyte growth factor (HGF), placenta growth factor (PlGF), angiogenin, angiopoietin (Ang)-1, interleukin (IL)-8, IL-6, IL-17, matrix metalloproteinases (MMPs), and tumor necrosis factor (TNF) stimulate angiogenesis. Inhibitors of neovascularization include angiostatin, endostatin, thrombospondin, tissue inhibitors of matrix metalloproteinases (TIMP), interferon (IFN)-α, IL-10, IL-12, IL-18, IL-23, IL-25, IL-27, and Ang-2. The various kinds of cells participating in the regulation of angiogenesis, mainly by secreting stimulatory and/or inhibitory factors, include endothelial cells, macrophages (MΦ), mast cells, fibroblasts, CD56^+^CD16^-^ natural killer (NK) cells, T cells, neutrophils, basophils, eosinophils, and dendritic cells (DCs) [7,14,15,16,17,18].

Angiogenesis is a complex and multi-stage process usually induced by hypoxia, which stimulates hypoxia-inducible factor-1 (HIF-1) leading to gene expression for proangiogenic factors, such as the most powerful stimulator, vascular endothelial growth factor (VEGF), and its receptor VEGFR-1/FLT-1 (Figure 1) [4,5]. Many different kinds of cells participate in the regulation of angiogenesis, mainly by secreting various factors which control this process (Figure 1). The diverse group of angiogenesis regulating cells includes natural killer (NK) cells, which are innate lymphocytes (generally cytotoxic ones) that provide protection against viral infections and tumor metastasis. NK cells also modulate other aspects of the immune system through the rapid production of numerous cytokines and chemokines. Out of the broad spectrum of NK cell subpopulations, some were shown to affect angiogenesis by VEGF, placenta growth factor (PlGF), interleukin (IL)-8/CXCL8, IL-10, angiopoietin (Ang)-1, and Ang-2 production [6]. There is a unique subset of decidual NK (dNK) cells known to participate in vascularization during embryonic and placental development [7]. Recently, a similar nurturing activity of NK cells has been described under pathological conditions, predominantly in different types of cancer, which indicates the relevance of NK cells in the development of ADDs [8,9,10,11,12,13]. A better understanding of the mechanisms in which NK cells regulate angiogenesis and its disorders may be of therapeutic significance. In this article, we will summarize the current body of knowledge on NK cell biology, with a special focus on the angiogenic properties of NK cells in cancer and other ADDs.

## 2. NK Cell Biology

NK cells constitute up to 15% of circulating lymphocytes and can be found in various organs and tissues. They are a subset of a heterogeneous family of innate lymphoid cells (ILCs) and originate from common lymphoid progenitors which derive from bone marrow hematopoietic stem cells (HSCs) [19].

NK cells are endowed with a variety of receptors, some inhibitory and some activating, which recognize human leukocyte antigen (HLA) class I, HLA class I-like, and non-HLA molecules on target cells. Integrated signals coming from both inhibitory and activating receptors regulate NK cell activity, which is manifested by the produced cytokines and cytotoxicity. Consistently, the combination of NK cell receptor ligands expressed on the target cells determines their life or death. In humans, the main inhibitory NK cell receptors consist of a big group of polygenic and polymorphic killer Ig-like receptors (KIRs) and heterodimeric CD94-associated lectin-like NKG2A receptors. Other inhibitory NK cell receptors, constitute a large and diverse group of molecules, including leukocyte immunoglobulin-like receptor subfamily B member 1 (LIRB1), NKR-P1A/CD161, programmed cell death protein 1 (PD-1), T-cell immunoreceptor with Ig and ITIM domains (TIGIT), CD96, CD112R, IL-1 receptor 8 (IL-1R8), T-cell immunoglobulin and mucin domain-3 (TIM-3), and lymphocyte activation gene 3 (LAG-3) [20,21,22,23,24,25,26,27,28]. The essential activating NK cell receptors are CD16, NKG2D, KIRs (with short cytoplasmic domains), and natural cytotoxicity receptors (NCRs), NKp30, NKp46, and NKp44 [29,30,31]. The activated state of NK cells is enhanced by many coreceptors, such as 2B4, NTB-A, DNAX accessory molecule-1 (DNAM-1/CD226), MAC-inhibitory protein (MAC-IP/CD59), and NKp80 [32,33,34,35,36].

Activated NK cells produce various cytokines, such as interferon (IFN)-γ, tumor necrosis factor (TNF), granulocyte-macrophage colony-stimulating factor (GM-CSF), IL-10, IL-5, IL-13, IL-22, chemokines, IL-8, macrophage inflammatory protein (MIP)-1α/CCL3, MIP-1β/CCL4, and RANTES/CCL5 [37,38]. To eliminate abnormal cells, NK cells use several mechanisms including induction of target cell apoptosis mediated by death receptors (FAS ligand- or TNF-induced apoptosis) or direct cytotoxicity through the release of perforin and granzyme granules [39,40,41]. Furthermore, NK cells can eliminate target cells via antibody-dependent cell-mediated cytotoxicity (ADCC) [42]. However, various cocktails of external factors may induce a substantial change in NK cell phenotype and function, causing NK cell differentiation into regulatory, memory, helper, and antigen-presenting cells [43,44,45]. Remarkably, mass cytometry analysis of human peripheral blood revealed the presence of approximately 30,000 phenotypes of circulating NK cells in a single individual [46]. These results clearly demonstrate an extreme and dynamic versatility of NK cells and indicate their significant and diverse role in the immune system.

### 2.1. NK Cell Subpopulations

Phenotypically NK cells are characterized by the expression of CD16 and CD56 surface molecules. There are two main subsets of peripheral blood NK (pNK) cells with different levels of CD56 expression and functional differences in cytokine production, the response to cytokines, and killing potential. The predominant CD56^dim^CD16^bright^ cells, which constitute up to 95% of pNK cells and are characterized by low expression of CD56, high level of CD16, and subsequent expression of CD94/NKG2A and KIRs, are considered mature. These cells are granular lymphocytes with a high cytotoxic potential. The second subpopulation, which is considerably smaller as it comprises up to 10% of pNK cells, shows high levels of CD56 and is known as the CD56^bright^CD16^−^ and CD56^bright^CD16^dim^ subset. These cells are considered immature, have low expression of CD16 and KIRs, and mostly lack granules. They localize mainly in lymphoid tissues, healthy solid organs, such as liver, kidney, and adrenal glands but also in various kinds of solid tumors [6,47,48]. Due to their strong sensitivity to stimuli, CD56^bright^CD16^dim^ cells show increased secretory activity and produce a variety of cytokines, such as IFN-ɣ, TNF, IL-5, IL-10, and IL-13 [49,50]. 

Populations of NK cells present in various tissues and organs, collectively called tissue-resident NK (trNK) cells, differ from the peripheral blood subsets in terms of their phenotypes and functions [51,52]. In general, CD56^dim^ cells predominate in the bone marrow, spleen, lung, breast tissue, and subcutaneous adipose tissue, while CD56^bright^ cells significantly predominate in the kidney, liver, thymus, adrenal gland, visceral adipose tissue, and in the gastric and intestinal mucosa-associated lymphoid tissue (GALT and MALT, respectively) [51,53,54,55]. 

#### dNK Cells

Another subpopulation of cells considered as tissue-specific are uterine/decidual NK cells. In healthy women, they are known to support endometrial spiral artery changes during the secretory phase of the menstrual cycle. Endometrial NK cells constitute 30–40% of uterine lymphocytes in the proliferative phase and up to 70% in the secretory phase [56,57,58]. Uterine NK (uNK) cells of non-pregnant woman are CD56^+^CD16^−^ and show reduced cytotoxicity, due to fewer activating NKp30 and NKp44 receptors [59]. Proangiogenic properties of uNK cells are manifested by the production of VEGF, angiogenin, and fibroblast growth factor (FGF)b, as was estimated in the menstrual cycle 7 days after the luteinizing hormone surge [55,60,61,62,63,64,65]. Recently, Dong et al. reported the expression of the proangiogenic marker ephrin-B2 on mouse uNK cells, which supported their contribution to spiral artery remodeling [66]. 

Over the course of pregnancy, dNK cells are essential for proper embryonic development. During the first trimester, dNK cells constitute 50–70% of decidual infiltrating lymphocytes, ensuring the induction and maintenance of immune tolerance at the maternal-fetal interface. Unlike pNK cells, dNK cells have low cytotoxic and mainly immunoregulatory (predominantly immunosuppressive) properties [67]. The phenotype of dNK cells is characterized by CD56^bright^CD16^-^KIR^+^CD9^+^CD49^+^ expression [68,69]. CD9 is an integrin-binding surface antigen belonging to the tetraspanin family, while CD49a is a α1β1 integrin receptor α-subunit (VLA-1). Both molecules are associated with the migratory and invasive activity of NK cells [13]. Recently, in a single-cell reconstruction study of the maternal-fetal interface, Vento-Tormo et al. identified three distinctive dNK subpopulations, namely: dNK1, dNK2, and dNK3 cells, all sharing *CD49A* and *CD9* tissue markers [70]. The dNK1 cells are characterized by the expression of *CD39* (ectonucleoside triphosphate diphosphohydrolase-1), *CYP26A1* (member of cytochrome P450 enzymes superfamily) and *B4GALNT1* (beta-1,4-N-acetylgalactosaminyltransferase 1) and higher levels of mRNA for granzymes. The dNK2 cells have *ANXA1* (annexin A1) and *ITGB2* (integrin subunit beta 2/CD18) markers, whereas the dNK3 cells display *CD160*, *KLRB1* (CD161), and *CD103* (integrin subunit alpha E) markers. Each of these subsets exhibited an absence of significant VEGF production, which was probably due to a lack of stimulation with IL-2 or IL-15.

During the course of pregnancy, dNK cells support decidual vascularization and the development of spiral arteries, which enhances the nutritional role of the placenta. The strong proangiogenic effect of dNK cells is manifested by the secretion of many proangiogenic cytokines and chemokines, such as VEGF, IL-8, angiogenin, Ang-2, stromal-derived factor-1 (SDF-1/CXCL12), IFN-γ, as well as matrix metalloproteinase (MMP)9 and MMP2 [6,9,55,58,71]. It is particularly metalloproteinases that stimulate angiogenesis and trophoblast invasion through extracellular matrix (ECM) remodeling [72]. The proangiogenic and cytotoxic profiles of dNK cells in comparison with those of pNK and decidual-like tumor-infiltrating and tumor-associated NK (TINK and TANK, respectively) cells is presented in Table 1.

The uterine microenvironment (UME) has strong immunosuppressive and proangiogenic properties. The distinctive phenotype of dNK cells is a result of a unique combination of cytokines, chemokines, and cell-to-cell interactions [68]. The UME is rich in transforming growth factor (TGF)-β, glycodelin A (GdA), and galectin and is characterized by prevalent hypoxia. In vitro studies on the effects of TGF-β on pNK cells have demonstrated induction of proangiogenic phenotype in treated NK cells. This phenotype was manifested by an increased migration and expression of chemokine receptors (CXCR3 and CXCR4) and invasion markers, such as, CD9, CD49a, and CD103; as well as VEGF secretion and a reduction of cytotoxic properties [9,13]. Interestingly, the effect of TGF-β was enhanced by IL-15 and IL-18, which are generally considered to be stimulatory factors of NK cells [73]. However, IL-15 was also found to have inhibitory properties, as it was shown to downregulate NKp44 and NKG2D expression and granzyme B production by NK cells in the course of endometriosis [74]. Hypoxic conditions ensure an increased expression of proangiogenic *VEGFA*, *CXCL8*, *CXCR4*, and *CXCR3* [75]. The lipocalin GdA, which is highly concentrated in the decidua, participates in fetomaternal defense and placental development. It modulates the differentiation and activity of several decidual immune cell types, such as macrophages, lymphocytes, and dendritic cells (DCs), providing fetomaternal tolerance [76]. It also enhances the proangiogenic properties of NK cells and the secretion of VEGF [77]. The proangiogenic galectin-1 works in a similar way and supports the maintenance of decidual immune surveillance [77]. Vacca et al. suggest that dNK cells cooperate with the neutrophils, myeloid cells (monocytes and macrophages), T lymphocytes, and stromal cells present in the decidua [68]. In the early stages of pregnancy, they interact with each other via cell-to-cell dependent or independent mechanisms to regulate the innate and adaptive immune response. 

## 3. Modulatory Role of Tumor Microenvironment (TME) in NK Cell Proangiogenic Activity

The TME is a specific niche defined as a cellular environment where tumor and cancer stem cells are embedded. It consists of the surrounding immune cells, endothelial cells, ECM, fibroblasts, bone marrow-derived inflammatory cells, and signaling molecules, which create a network of mutual influences with cancer cells [85,86]. Neoplastic tissue imposes a multidimensional tumorigenic and angiogenic program on TME components, which results in a decreased antitumor killing potential of immune cells, state of anergy, and acquisition of proangiogenic properties [9,87]. This program involves immune cells, inflammatory cells, and tumor-associated stromal cells and their bioactive products, such as cytokines, growth factors, ECM, and secreted microbubbles [88,89,90]. The immunosuppression and induction of angiogenesis that occur during tumor development are similar to those occurring in the uterus/decidua during the first weeks of pregnancy (Table 1).

### 3.1. The Effect of TME Bioactive Components and Hypoxia on NK Cells

**Figure 2:**
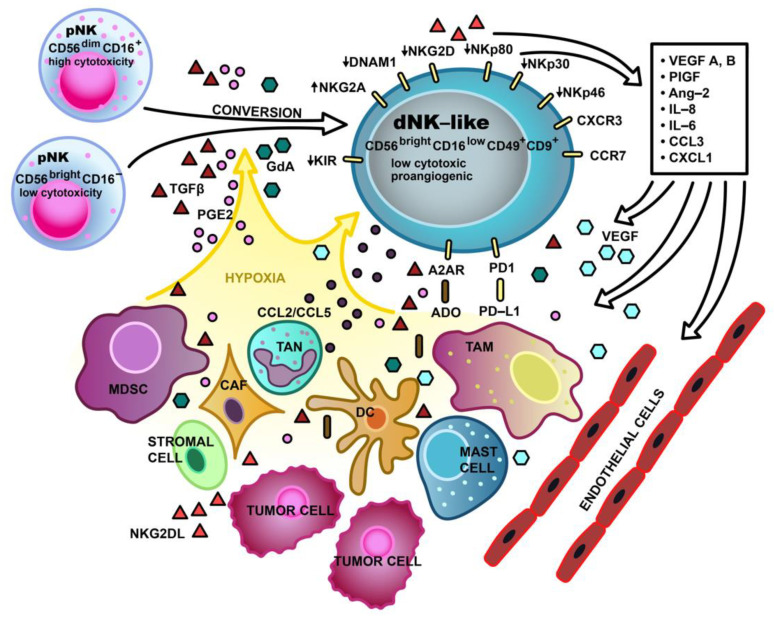
Proangiogenic phenotype of tumor-infiltrating natural killer (TINK) cells induced and maintained by the tumor microenvironment (TME). The TME induces phenotype conversion of highly cytotoxic CD56^low^CD16^bright^ and weakly cytotoxic CD56^bright^CD16^−^ natural killer (NK) cells derived from peripheral blood (pNK) to CD56^bright^CD16^low^CD49^+^CD9^+^ decidual-like NK (dNK-like) cells. Cellular components of TME, namely tumor-associated macrophages (TAM), cancer-associated fibroblasts (CAF), tumor-associated neutrophils (TAN), myeloid-derived suppressor cells (MDSC), DC, mast cells, cancer cells, and stromal cells, produce proangiogenic, and tolerogenic factors which affect TINK cells. The main factors participating in the pNK-cell phenotype switch are TGF-β, glycodelin-A (GdA), prostaglandin E2 (PGE2), and hypoxia. Moreover, these factors together with adenosine (ADO), PD-L1, and NKG2DL maintain the dNK-like phenotype of TINK cells. This weakly cytotoxic and proangiogenic phenotype is manifested by an increased NKG2A expression; decreased KIR, NKG2D, NKp30, NKp46, NKp80, and DNAM1 expression; presence of CXCR3 and CXCR7; and upregulated production of VEGF, PlGF, Ang-2, IL-6, IL-8, CCL3, and CXCL1. In turn, proangiogenic factors produced by TINK cells affect certain cellular components of the TME, including endothelial cells, and stimulate neovascularization.

The availability of activating and inhibiting cytokines in TME is crucial for the regulation of NK cell functions [91]. The most powerful activating cytokine is IL-15, with CD4^+^ T cell-derived IL-2 and the type I IFNs that bind to toll-like receptors also having a strong stimulating effect on NK cells. Furthermore, immunostimulatory cytokines include IL-21, IL-27, and IL-18. However, IL-18 was also shown to act synergistically with TGF-β and impair NK recruitment and killing capability. The activity of NK cells is downregulated by immunosuppressive factors, such as TGF-β or adenosine (ADO), which affect NK cell maturation and cytotoxicity [92,93], as illustrated in Figure 2. Moreover, some cytokines produced in the TME may act indirectly by recruiting suppressive cells, for example, TGF-β-dependent regulatory T lymphocytes [92].

#### 3.1.1. TGF-β

TGF-β is the most immunosuppressive cytokine in the TME and its increased expression has been found in many types of tumors [94]. Elevated levels of this cytokine were also found in the plasma of patients with lung cancer [95], colorectal cancer (CRC) [96], and renal cell carcinoma (RCC) [12]. Various tumor cells, including cancer cells, stromal cells, immune cells, and myeloid-derived suppressor cells (MDSCs) are responsible for the excessive TGF-β production in cancer patients [97]. In order to restrict innate immune surveillance of NK cells in the TME, TGF-β induces a conversion of NK cells to intermediate type 1 ILCs. Due to the non-canonical TGF-β signaling, these cells express CD69, CD49a, and *Tnfsf10* (a member of the TNF superfamily) and lose the expression of T-box transcription factor Eomesodermin (EOMES) [98].

TGF-β1 was also demonstrated to convert the pNK cells of healthy donors to cells of a dNK-like phenotype [99,100]. Bruno et al. revealed that cytolytic CD56^+^CD16^-^ NK cells from the peripheral blood of healthy subjects exposed to TGF-β1 display enhanced production of VEGF and PlGF [8]. According to the authors, TGF-β participates in proangiogenic changes of NK cells, which acquire a decidual-like phenotype manifested by CD56^bright^CD16^-^ CD9^+^ and/or CD49^+^ expression (Figure 2). As mentioned above, CD9 and CD49a are the markers of proangiogenic dNK cells and are not expressed by pNK cells [99].

Cardeira et al. showed that TGF-β induces CD9 expression on pNK cells isolated from healthy subjects, and the level of CD9 increases when pNK cells are cultured with a combination of hypoxia and TGF-β1 [99]. Guan et al. confirmed the participation of TGF-β, along with other factors, such as hypoxia, in the conversion of pNK-like cells to a dNK-like phenotype in both decidua and cancer [12]. Subsets of NK cells with a dNK-like phenotype have been found in peripheral blood, pleural effusion, and in various types of cancer, such as CRC and lung cancer [8,9,11,82]. TGF-β was also shown to enhance the expression of CXCR3 and CXCR4, which is characteristic of dNK cells, in TINK cells [13]. The CXCR3 marker was expressed in melanoma, breast cancer and glioma, whereas CXCR4 was present in lung cancer and CRC. Interestingly, TGF-β is associated with the epithelial-to-mesenchymal transition (EMT), a process by which cancer cells metastasize [9,11,72,101,102]. 

Except for proangiogenic changes, TGF-β also causes NK cell dysfunction. This is manifested by a reduced killing potential and decreased granzyme and perforin secretion, as was demonstrated in many types of cancer, including breast cancer and hepatocellular carcinoma [103,104]. TGF-β1 suppresses NK cell-activating receptors, such as NKG2D, in patients with prostate and other cancers [105,106]. This lowered expression of NKG2D receptors inhibits the ADCC of and the CD16-mediated IFN-ɣ production by human NK cells [104]. The loss of activating receptors, such as NKG2D, NKp30, NKp46, and DNAM-1, is positively correlated with tumor progression in gastric cancer [105]. TGF-β blockade retains the function of ex vivo activated and expanded NK cells in tumor models [107].

NK-cell-suppression by the TME (induced by TGF-β, hypoxia, etc.) may be partially explained by the inhibition of NK cell signaling via signal transducer and activator of transcription (STAT) proteins. In neoplasms, the Janus kinase (JAK)-STAT pathway changes and is constitutively active [108]. This pathway modulates many functions of NK cells and transduces signals from various cytokines. *STAT5* expression is essential for pNK cell survival, since deletion of both the *Stat5a* and *Stat5b* homologues leads to an apoptotic death of these cells [109]. STAT5 is involved in the downstream signaling of the powerful NK cell stimulators IL-2 and IL-15. Both STAT5A and STAT5B isoforms are known to be involved in NK cell maturation [109]. STAT5 also regulates NK cell proliferation and cytotoxicity [110]. Furthermore, STAT5B was shown to inhibit *Vegfa* expression in NK cells and hence contribute to the inhibition of angiogenesis and tumor growth [110]. STAT5B signaling inhibition in the TME may result in lost or weakened NK cell cytotoxic functions and enhanced NK cell proangiogenic activity, which, in turn, promote tumor growth and metastasis [92,110].

#### 3.1.2. Hypoxia

The hypoxic environment, which is present in the decidua and in various types of tumors, is known to support angiogenesis. As already mentioned, under hypoxic conditions, HIF-1 transcription factors are activated and induce the expression of proangiogenic genes through the HIF-1α/VEGF axis [111]. In neoplasms, hypoxia affects cancer, immune, and stromal cells which start to produce cytokines stimulating angiogenesis, vascular development, and tumor progression. Hypoxia also changes the phenotype of NK cells (Figure 2). After one week of in vitro hypoxic (1% O_2_) culture of pNK cells from healthy donors, the cultures were richer in CD56^bright^CD16^−^ cells, which secreted VEGF-A, than cultures obtained under 21% O_2_. Supernatants from these cultures exhibited proangiogenic potential, as was estimated by HUVEC cell angiogenic activity [99]. Guan et al. studied the phenotypes and functions of pNK cells and TINK cells originating from RCC patients [12]. The authors found that the conversion of the pNK to dNK-like phenotype in RCC was favored by hypoxia. The pNK cells of RCC patients mostly had a CD56^+^CD16^bright^ phenotype and decreased cytotoxic potential. Also in this case, when cultured under hypoxic conditions (1% O2), pNK cells showed an increased VEGF production and an even lower cytotoxicity potential compared with normoxic cell cultures. Furthermore, TINK cells showed the phenotypic features of dNK-like CD56^+^CD16^dim/-^ cells and an increased expression of proangiogenic genes and inflammatory factors via *HIF1A* pathway gene activation. Krzywinska et al. demonstrated that *Hif1a* deletion in mouse NK cells resulted in reduced tumor growth due to non-productive angiogenesis manifested by numerous immature vessels, hemorrhages, and severe hypoxia, which favored metastasis [112]. The authors identified the hypoxic response of NK cells as an inhibitor of VEGF-driven angiogenesis. HIF-1α appears to be responsible for reduced bioavailability of VEGF owing to the expression of soluble VEGFR-1 (sVEGFR-1). This mechanism of lowering excessive amounts of VEGF allows for the formation of functionally mature vessels, which promotes tumor growth.

#### 3.1.3. GdA

As mentioned above, GdA is involved in the development of immune tolerance during pregnancy and regulation of normal reproductive functions. GdA is also found in a variety of malignancies, including endometrial, ovarian, breast, lung, and rectal cancer [77]. Lee et al. observed that GdA interacts with L-selectin, which was found on CD56^bright^CD16^−^ NK cells. Consequently, CD56^bright^CD16^−^ NK cells bind GdA more strongly than CD56^dim^CD16^+^ NK cells [113]. In the same study, the authors showed an increase in the expression of CD9 and CD49a markers, as well as an enhanced production of ERK-dependent VEGF by CD56^bright^CD16^−^ pNK cells treated with GdA. Furthermore, the supernatants from these cultures had a proangiogenic effect on HUVEC cells and stimulated trophoblast invasion by inducing the production of insulin-like growth factor-binding protein 1 (IGFBP-1). To summarize, GdA plays a role in transforming CD56^bright^CD16^−/low^ cells into dNK-like cells capable of producing VEGF (Figure 2) [113].

#### 3.1.4. Galectins

Galectins are β-galactoside-binding proteins, which affect NK cell function and are highly expressed in certain types of cancer. Galectin-1 is present on NK cells and is also known to be overexpressed in glioma, melanoma, myeloma, breast, and ovarian cancer [114,115,116]. High levels of galectin-1 in glioma have been associated with inhibiting NK cell cytotoxicity. Galectin-1-deficient mice had reduced tumor growth, increased NK cell infiltration, and elevated granzyme B expression proving an inhibitory effect of galectin-1 on NK cell antitumor function [117]. Moreover, galectin-1 has a stimulating effect on angiogenesis, since specific galectin-1-neutralizing antibodies were shown to suppress angiogenesis and tumor formation in vivo [13]. Another member of galectin family, galectin-3, is highly expressed in tumors and is also associated with angiogenesis. NK cells differ in the expression of galectin-3, whose presence was detected both in their cytoplasm and nuclei, but not on the surface of their cell membranes [118,119]. Functionally, galectin-3 correlates with the degree of NK cell degranulation and has been suggested to inhibit NK cell immunosurveillance. Due to the possible therapeutic potential of galectin-1 and -3 inhibitors, there is an ongoing clinical trial on their use in the treatment of certain types of cancer [115].

#### 3.1.5. Prostaglandin E2 (PGE2) 

PGE2, which is present in high concentrations in tumors, promotes their growth, development, and angiogenesis [87]. PGE2 inhibits NK cell functions, negatively affecting NKG2D, NCRs, and ADCC. According to Bassani et al., it is probable that PGE2 is involved in the development of the angiogenic and anergic phenotype of NK cells in tumors (Figure 2) [87]. 

#### 3.1.6. ADO

ADO is a metabolite generated from ATP by CD39 and CD73 under hypoxic and extracellular stress conditions. ADO levels increase during decidualization and in the extracellular fluid of solid carcinomas [87]. ADO exhibits immunosuppressive activity, which regulates an excessive immune reaction during inflammation and tissue damage [120,121]. It negatively influences CD8^+^ T cell, macrophage, and NK cell function and TME infiltration. Prestimulated with IL-12 and IL-15 and exposed to ADO, NK cells enhance IFN-γ expression in the CD56^bright^ and CD56^dim^ subpopulations. ADO negatively influences the activating receptors NKG2D and NKp30 of NK cells [122]. Young et al. show that ADO decreases the maturation process of NK cells via A2AR receptors (Figure 2) [121]. 

#### 3.1.7. HLA-G

HLA-G is a nonclassical MHC class I molecule, the expression of which indicates poor prognosis in numerous neoplasms [87]. One of the effects of HLA-G is to inhibit NK cell activity by interacting with KIR2DL4, which induces proinflammatory and proangiogenic cytokine production [123].

### 3.2. TME Cellular Components and Their Effect on NK Cells

#### 3.2.1. Cancer Cells

Cancer cells cause anergy of NK cells by secreting a number of immunosuppressive factors, including IL-10, TGF-β, PGE2, ADO, and two tumor enzymes: arginase 1 (Arg1) and indolamine 2,3-dioxygenase (IDO) [124]. Moreover, cancer cells can release soluble NKG2D ligands (NKG2DL), which mask NKG2D receptors or reduce their activity on NK cells. 

Apart from cancer and immune cells, TGF-β is also produced by the platelets that cover tumor cells and enhance the TGF-β effect exerted by TME cells. Furthermore, cancer cells affect NK cells indirectly by regulating activity of other cellular TME players, which in turn may interact with NK cells in a direct cell-cell contact-dependent manner or through secreted cytokines, chemokines, and other factors [125,126].

#### 3.2.2. Tumor-Associated Neutrophils (TANs)

TANs are a subset of neutrophils with acquired tumor-promoting and proangiogenic properties. They are responsible for the inhibition of NK cell cytotoxic functions by secreting reactive oxygen species (ROS) and Arg1 [127,128]. However, this only applies to CD16^+^ NK cells, because, probably due to its antioxidative properties, the CD56^bright^CD16^−^ subset is resistant to ROS. Therefore, it is believed that TANs may contribute to the expansion of the proangiogenic CD56^bright^CD16^−^ subpopulation [129]. Owing to the secretion of chemokines, namely, CCL2 and CCL5, TANs can recruit NK cells to the tumor site and, vice versa, NK cells attract neutrophils by secreting CXCL8 [130,131]. Although, NK cells contribute to increased angiogenesis by attracting neutrophils, they also display inhibitory properties towards TANs. The results of Ogura’s studies indicate that NK cells control angiogenic and tumor-promoting functions of TANs through the release of IFN-ɣ and through the IL-17A axis. In NK cell-deficient mice, TANs showed increased angiogenic activity resulting in tumor growth promotion [129].

#### 3.2.3. Mast Cells

Mast cells are bone marrow-derived, long-living population of connective tissue-resident cells. Immature mast cells circulate in the blood. Being involved in antibacterial and antiviral response, as well as in immunomodulatory processes, mature mast cells play a critical role in both the innate and adaptive immunity. Mast cells actively participate in tumor development by producing many proangiogenic factors, such as bFGF, VEGF, IL-8, TNF, TGF-β, and nerve growth factor (NGF). They induce angiogenesis, as demonstrated in functional in vitro models, including chick embryo chorioallantoic membrane assays [7,16,132]. An increased concentration of mast cells was noted in both hematological neoplasms and solid tumors [132]. Ribatti et al. hypothesize that mast cells may synergistically interact with NK cells in the development of tumor angiogenesis and thus promote cancer progression [7]. Mast cell-derived TGF-β participates in the polarization of the CD56^dim^CD16^+^ NK population towards a proangiogenic CD56^bright^CD16^−^ phenotype. Moreover, mast cells suppress NK cell cytotoxic activity by releasing ADO, which inhibits NKG2D and NKp30 receptors and IFN-ɣ production [87].

#### 3.2.4. Macrophages

Macrophages, generally, polarize into two groups, classically activated M1 cells and alternatively activated M2 cells. Tumor-associated macrophages (TAMs) have features of the M2 macrophages and promote angiogenesis in tumors by secreting proangiogenic cytokines (e.g., VEGF, epidermal growth factor [EGF], FGFs and Ang-1), MMP proteases, plasmin, and plasminogen activator [133,134]. TGF-β and PGE2, which are also produced by TAMs, promote the angiogenic phenotype of the CD56^bright^CD16^−^ NK cells. Furthermore, PD-L1 expressed by TAMs may bind to PD-1 present on NK cells and inhibit NK cell antitumor activity [87].

#### 3.2.5. Cancer-Associated Fibroblasts (CAFs)

CAFs perform several functions that support tumor growth, such as secretion of VEGF, FGF, platelet-derived growth factor (PDGF), and other proangiogenic factors. Like in the case of mast cell- and TAM-derived TGF-β, the TGF-β secreted by CAFs contributes to NK cell polarization towards the dNK-like phenotype, which, in turn, supports the process of EMT [102,135]. CAFs and TAMs reduce the cytotoxic functions of NK cells by downregulating the expression of NKG2D receptors, NCRs, and DNAM-activating receptors, and reducing the release of IFN-ɣ, granzymes, and perforins [136]. Furthermore, CAFs also produce PGE2, which participates in NK cell suppression [8,137].

#### 3.2.6. MDSCs

MDSCs are a heterogeneous group of immature cells, comprising two distinct subpopulations. One is the monocyte-like M-MDSC subset and the other is the polymorphonuclear-like MDSC (PMN-MDSC) subpopulation. Within the TME, MDSCs secrete a number of proangiogenic cytokines (e.g., VEGF, MMP9, bFGF) and participate in the conversion of endothelial cells [137,138,139]. MDSCs inhibit the activity of both NK cells and cytotoxic T cells [140,141]. MDSCs downregulate the cytotoxic functions of NK cells through direct contact (PD-L1 expression) and production of soluble factors, such as TGF-β, ADO, and PGE2. MDSCs target NCR activation, including NKp30, by affecting the CD247 subunit [142,143]. The inhibition of the NK cell cytotoxic function is manifested by lower degranulation capacity and decreased IFN-ɣ secretion [143].

#### 3.2.7. DCs

DCs infiltrate the TME in response to β-defensins released by the tumor [144]. The tumor-infiltrating DCs that are adversely affected by the TME do not fulfill their main functions (antigen presentation). Instead, they undergo incomplete differentiation and activation and are gradually transformed into suppressor cells affecting NK cells, effector T lymphocytes and regulatory T cells. Consequently, further development and growth of the neoplasm is promoted [145,146]. DCs produce immunosuppressive factors, such as cytokines (IL-10, TGF-β, IL-6, IL-8), Arg1, IDO, PGE2, NO, and ADO, which significantly affect the function of NK cells and other components of the TME [72,145].

The effect of different TME components on the NK cell phenotype is graphically presented in Figure 2.

## 4. Proangiogenic Potential of NK Cells in Cancer

Since NK cells are very efficient at killing cancer cells (especially the circulating ones) and limiting tumor metastases, individuals with low NK cell activity show a significantly higher risk of neoplastic disease [147,148]. Subsequently, cancer progression is correlated with low numbers and impaired cytotoxic activity of TANKs and their impaired ability to release proangiogenic and immunosuppressive factors [149,150,151,152]. A growing body of data shows that the phenotypical and functional change in circulating and TINK cells is a result of unique, cancer-associated conditions described above (Figure 2). Within solid tumors, the potentially cytotoxic NK cells undergo anergy characterized by activating receptor downregulation, reduced degranulation, and impaired abilities to release perforin, granzyme, and anti-tumor cytokines [153,154,155,156,157]. The factors contributing to NK cell anergy in the TME include an extremely poor access to nutrients, such as glucose and glutamine [158]. Since NK cell activation is highly dependent on the metabolic pathway of glycolysis, tumor-driven glucose restriction leads to an impaired NK cell antitumor function [159]. Accumulation of proangiogenic NK cells in the TME may be a result of a few different mechanisms. One of them is the TME-mediated reprogramming of NK cells described above. Another one is selective recruitment of CD56^brigt^CD16^−^ cells and their local decidualization (Figure 2). The stress factors and cytokines released within tumors reduce the production of chemokines responsible for the recruitment of CD56^dim^ NK cells (CXCL2, CX3CL1, CXCL1, and CXCL8) and increase production of those responsible for attracting CD56^bright^ NK cells (CCL2, CXCL9, CXCL10, and CCL5) [48,160,161]. According to a very recent paper by Riggan et al., two chemokine receptors, CCR2 and CXCR3, typically expressed by the CD56^bright^ subpopulation are particularly involved in the trafficking of NK cells to tumors [162].

As already mentioned, different types of cancer can affect pNK cell population composition. A large proportion of CD56^brigt^CD16^dim/−^ NK cells have been observed in the peripheral blood of CRC, lung and breast cancer patients. Other findings included a diminished expression of activating receptors, higher expression of inhibitory NK cell receptors, and lower cytotoxic abilities of these cells [8,80,81]. In other types of cancer, even if the proportion of circulating CD56^dim^CD16^bright^ cell subset remained stable, its cytolytic abilities were reduced [12]. Furthermore, the pNK cells of non-small cell lung cancer (NSCLC), CRC, and melanoma were found to produce elevated levels of proangiogenic factors [9,72,163]. The pNK cells of patients with acute myeloid leukemia (AML) were reported to show impaired cytolytic functions and significant underexpression of cytotoxic NKp30, NKp44, and NKp46 receptors [164]. 

A significant increase in the proportion of tumor-infiltrating CD56^brigt^CD16^low^ cells has been reported in many different types of cancer, such as lung, breast, CRC, melanoma, RCC, head and neck squamous carcinoma, and hepatocellular carcinoma [8,10,11,80,81,83,90,165,166,167]. These tumor-infiltrating cells have low levels of perforin [8,10,90] and granzyme, decreased expression of NKp30, NKp80, NKG2D, and DNAM1 [168], and elevated expression of NKG2A [80,169]. Furthermore, as already mentioned, a rapidly growing number of studies report proangiogenic properties of tumor-infiltrating CD56^bright^CD16^dim^ cells and indicate that they share the phenotype and functions with dNK cells (Figure 2) [8,10,11,13,82,87].

### dNK-Like Cells in Cancer

Among different cellular TME antiheroes, like neutrophils, macrophages, mast cells, or fibroblasts, which support angiogenesis and cancer development, dNK-like cells rise as a very important subset of players. As mentioned earlier, the decidual-like phenotype of poorly cytotoxic CD56^bright^CD16^−^ NK cells is promoted by the local hypoxic conditions and specific cocktail of factors produced by the TME and the tumor itself (Figure 2). Albini and Noonan hypothesize that tumors use the nurturing potential of NK cells and through mimicking fetal growth “polarize NK cells toward CD56^bright^, poorly cytotoxic, pro-proliferative and proangiogenic CD9-positive cells” [13]. Upon chronic exposure to TGF-β, distinct circulatory subsets of NK cells were shown to display a plasticity characteristic of ILCs and to adopt an ILC1 phenotype [170,171]. This change is manifested by the expression of tissue residency markers, CD9, CD49a, and CD103, and reduced cytotoxic abilities of the converted cells.

The presence of dNK-like cells has been recently reported in lung, colorectal, and breast cancer and in melanoma, renal cell, and glioblastoma tumors (Table 1) [9,11,12,72]. One study on breast carcinomas, melanoma, and colon cancer tissues, where the majority of TINK cells belonged to the CD56^bright^CD16^dim^ subset, demonstrated high expression of dNK cell markers CD9 and CXCR3 in the breast and colon tumors [11]. Additionally, sections from colon and breast carcinomas showed the majority of immunostained CD56-positive cells to be positive for VEGF. Bruno et al., who identified CD56^bright^CD16^−^ cells as the predominant tumor subset of NK cells in NSCLC, also demonstrated their association with VEGF, PlGF, and IL-8 production [8]. Furthermore, functional in vitro assays showed proangiogenic potential of supernatants collected from NSCLC CD56^+^CD16^−^ NK cell cultures, which induced endothelial cell chemotaxis and capillary-like structure formation.

Lately, the same group compared TINK and TANK cells derived from CRC patients with control NK cells of patients with bowel disease characterized by non-oncological inflammation [10]. The authors demonstrated that TINK/TANK cells exhibit the dNK-like CD56^bright^CD16^dim/−^CD9^+^CD49^+^ phenotype and concomitantly decreased expression of NKG2D (Table 1). This was accompanied by a failure of functional cytotoxic activity manifested by reduced degranulation. TANK proangiogenic activity was indicated by the secretome of those cells, which included VEGF, angiogenin, and ECM-degrading enzymes, such as MMP2/9 and tissue inhibitor of matrix metalloproteinases (TIMP). Indeed, functional in vitro assays revealed TINK and TANK cell ability to induce proliferation, migration, and adhesion of endothelial cells, which resulted in the formation of capillary-like structures. The angiogenic switch of TINK and TANK cells occurred through STAT3/STAT5 pathway activation. Administration of the STAT5 inhibitor pimozide inhibited most of the angiogenic parameters, such as secretion of VEGF and angiogenin, as well as the formation of vascular structures. The pattern of TIMP1, TIMP2, and MMP9 expression did not change under the influence of pimozide. These results contradict those previously described by Gotthardt et al., who showed STAT5B to be responsible for the inhibition of *Vegf* expression [110]. Therefore, it is plausible that STAT5A and STAT5B play opposite roles in the regulation of VEGF production by NK cells.

Another, already mentioned study by Guan et al. confirmed TINK cell conversion to a dNK-like phenotype in RCC, where a significantly larger subset of CD56^+^CD16^dim/−^ was present (Table 1) [12]. These cells expressed nine genes (encoding VEGF-A, VEGF-B, Ang-2, IL-6, IL-8, CCL3, CXCL1, CCR7 and CD146 receptor), with the level of their expression similar to that observed in dNK cells and notably higher than that observed in pNK cells (Figure 2). All of these factors participate in angiogenic, homing, and/or immunosuppressive mechanisms, which contribute to cancer progression and metastasis. One study involving a xenograft model of gastric cancer demonstrated CXCL1 potential to increase VEGF expression, microvessel density, and local tumor growth [172]. Similarly, CCL3 promotes VEGF expression in human osteosarcoma cells and subsequently induces tube formation by human endothelial progenitor cells [173]. Furthermore, a recent study on human esophageal squamous cell carcinoma in a nude mouse xenograft model revealed strong angiogenic capacity of the CCR7 receptor [174]. Cai et al. demonstrated a significant inhibition of tumor growth in animals vaccinated with iRNA targeting CCR7 and subsequent downregulation of protein expression for VEGF-A, VEGF-C, IL-6, IL-8, TNF, TGF-β, and NF-κB. Finally, CD146, also known as the melanoma cell adhesion molecule is, in fact, a potent receptor of growth factors, proangiogenic factor receptors and ECM components, such as galectins [175]. CD146 is implicated in tumor progression due to vascular and lymphatic vessels pathological development. CD146 overexpression was reported in the majority of cancer types in primary lesions, as well as in metastases [175,176].

Decidual-like proangiogenic polarization of the NK cells was also reported in malignant pleural effusion (PE) by Bosi et al. (Table 1) [82]. The study revealed an acquired CD56^bright^CD16^−^ phenotype of NK cells obtained from pleural fluids of patients with inflammatory conditions, primary, and metastatic tumors. PE-derived NK cells from all these groups were characterized by reduced cytotoxicity manifested by impaired degranulation capacity and reduced perforin release. Both the patients with metastatic tumors and those with primary tumors showed an increased expression of CD49a and CD69 and decreased expression of maturation marker CD57, which are characteristic of the dNK-like cell phenotype. The authors also found increased production of proangiogenic VEGF by PE-derived NK cells isolated from metastatic cancer patients. Moreover, supernatants from purified NK cells derived from the PE of patients with metastasis stimulated endothelial cell angiogenesis in in-vitro morphogenesis tests. 

A recently published study by Gallazi et al. [84] demonstrated that prostate cancer TANK cells show a CD56^bright^CD9^+^CD49a^+^CXCR4^+^ phenotype and express PD-1 and TIM 3 (Table 1). These cells are characterized by an impaired cytotoxic function, as evidenced by a decrease in their degranulation capacity. They also release proangiogenic factors that induce angiogenesis in an in vitro model of inflammatory angiogenesis and increase expression of *CXCL8*, *ICAM1* (intercellular adhesion molecule), and *VCAM1* (vascular cell adhesion molecule) mRNA in endothelial cells. The studied subset of NK cells can also recruit THP-1 (human monocytic leukemia cell line) and peripheral blood CD14^+^ monocyte-derived macrophages and polarize them towards proangiogenic M2-like/TAMs [84].

Accumulation of tumor-associated CD49a^+^ NK cells was documented in hepatocellular carcinoma (HCC) [177]. In healthy individuals, CD49a^+^ NK cells are considered to be a liver-resident subset [178,179]. However, Sun et al. [177] observed that the higher numbers of CD49a^+^ in HCC were correlated with poor clinical outcomes. Flow cytometry analysis of tumor-infiltrating CD49a^+^ NK cells revealed an upregulated expression of inhibitory receptors and exhaustion-related checkpoint molecules such as PD-1, TIGIT, and CD96. Also, a significant increase in gene expression for TGF-β, IFN-γ, and TNF, with subsequent decrease in IL-15 and IL-18 levels were found. These results strongly suggest a negative regulatory function of the intratumoral CD49a^+^ NK cells and their possible proangiogenic potential. Very recently, Wu et al. confirmed a high correlation between NK cell scores and genes encoding immune checkpoint proteins, such as inhibitory KLRD1, PD-1, CD96, TIGIT, and CTLA-4 in HCC samples [151]. Thus, these molecules, once again, were indicated as potentially good immunotherapeutic targets. 

## 5. Proangiogenic Potential of NK Cells in Angiogenesis-Dependent Diseases

### 5.1. Endometriosis

Endometriosis is a chronic, inflammatory, gynecological, hormone-dependent disease. Its definition includes the presence of the endometrium-like tissue (glands and stroma) outside the uterine cavity, predominantly within the peritoneal cavity. The main symptoms of endometriosis include chronic pelvic pains, excessive menstrual cramps, pain during intercourse, subfertility, and infertility [56]. Development of endometriosis depends on immunological, hormonal, genetic, and environmental factors. Neovascularization plays a role in the implantation, growth, and survival of endometriotic lesions in the ectopic environment [57].

Eutopic endometrium of women with endometriosis has been reported to have disturbed angiogenic activity, as indicated by increased endothelial cell proliferation, higher microvessel density, and elevated levels of *VEGFA*, *ANGPT1* and *ANGT2* (Ang-1 and –2) mRNA, in comparison with that of healthy women [180,181]. The neovascularization of endometrial lesions has been associated with VEGF and other proangiogenic factors including TGF, TGF-β, bFGF, Ang, and hepatocyte growth factor (HGF) [180,182]. Moreover, recent studies emphasize the role of the Notch signaling pathway in sprouting angiogenesis of endometriotic lesions [183]. 

The peritoneal fluid (PF) of endometriosis patients was also described to have elevated concentration of proangiogenic factors, such as angiogenin, epithelial-neutrophil-activating peptide (ENA-78), erythropoietin, HGF, IGF-1, and VEGF, and a reduced concentration of antiangiogenic factors, adiponectin, IP-10 (CXCL10), and sVEGFR, which all together favor endometrial lesion development [184,185,186]. Furthermore, higher levels of TGF-β in the PF of endometriosis patients, in comparison with those of healthy subjects, have been recently reported [58,187,188]. TGF-β was related to the reduced cytotoxicity of NK cells in endometriosis. The cytotoxicity of NK cells isolated from ectopic endometrium is lower than of those from eutopic endometrium and healthy controls [189]. This may be a result of reduced NKG2D ligands expression [190].

In terms of angiogenesis markers and the production of proangiogenic cytokines, uNK cells of endometriosis patients are poorly described. It is plausible, that similarly to cancer and decidua, the local microenvironment of endometrial lesions (e.g., steroid hormones, chemokines, and cytokines, such as TGF-β) strengthens proangiogenic properties of uNK cells. 

Giuliani et al. demonstrated that eutopic endometrium in endometriosis patients comprises more immature NK cells (CD16- and NKp46-negative) compared with that in healthy women [191]. Therefore, fertility disorders associated with endometriosis may be the result of improper NK cell maturation. Furthermore, it was shown that the number of immature NK cells increases in ectopic endometrium in endometriosis [189]. Surgical removal of endometrial lesions increases the proportion of differentiated NK cells. NK cell maturation can be restored by an in vitro addition of stem cell factor [57,189,192]. To summarize, in endometriosis, less cytotoxic and more immature NK cells (with presumably proangiogenic features) are generated.

### 5.2. Eye Disorders—AMD and Corneal Neovascularization

Pathological neovascularization is involved in the pathomechanism of corneal neovascularization and AMD through corneal and choroidal angiogenesis, respectively. Excessive angiogenesis in the eye is dangerous as can lead to blindness. In both pathologies, angiogenesis is known to be associated with high levels of VEGF and other angiogenic cytokines, like bFGF [193]. Lee et al. [194] observed that VEGF in the eye may be produced not only by activated endothelial cells but also macrophages. In vitro coculture assays revealed that NK cells stimulate macrophages to produce VEGF by releasing IFN-ɣ and thereby increasing endothelial cell proliferation [194]. In a mouse model of bFGF-induced corneal angiogenesis, the authors proved that depletion of corneal NK cells reduced both angiogenesis and macrophage infiltration. Moreover, expression of mRNA encoding VEGF-A, VEGF-C, and VEGFR3 was significantly decreased. Removal of NK cells also suppressed choroidal neovascularization, VEGF, and IFN-ɣ levels, as demonstrated in a mouse model of laser-induced choroidal angiogenesis [194]. These results indicate a potential role of NK cells in the pathogenesis of corneal neovascularization and AMD via angiogenesis modulation.

### 5.3. RA

RA is a chronic, inflammatory, autoimmune, systemic disease characterized by nonspecific symmetric arthritis, extra-articular lesions, and systemic complications, leading to disability and premature death [195,196]. Many reports indicate that RA belongs to ADDs and is characterized by an excessive level of pathological angiogenesis which correlates with synovial membrane inflammation in joints [196]. The excessive angiogenesis is caused by high levels of proangiogenic factors, such as VEGF, bFGF, PDGF, Ang-1, Ang-2, and Tie2 (angiopietin receptor), which are mainly produced by activated macrophages and fibroblasts in the affected joint. A large number of leukocytes are recruited to the affected joint. This increases metabolic demands and creates proangiogenic hypoxic conditions, promotes synovitis, as well as bone and cartilage destruction [195,196]. 

The role of NK cells in the pathomechanism of RA is still under investigation, but accumulation of these cells in inflamed joints has been demonstrated [197,198]. Several reports focused on the NK cell phenotype in RA patients [198,199,200]. Yamin et al. evaluated the function and phenotype of NK cells derived from peripheral blood and synovial fluid (SF) of study patients [198]. The patients were divided into 2 groups, non-deformative disease (NDRA) and deformative disease (DRA), depending on the RA severity. The authors reported a significant increase in pNK cell numbers in patients with severe DRA, when compared with those in healthy controls and in NDRA patients (25% and 10%, respectively). These results were confirmed by Lin’s studies [200]. A similar trend was observed in the SF, where NK cells accounted for 21% of all SF cells in DRA patients and only 6% in NDRA patients [198]. A high percentage of CD56^bright^ NK cells was noted in the SF of both DRA and NDRA patients (39.9% and 37.7%, respectively) [198]. NK cell cytotoxic activity was decreased in RA patients comparing with that in healthy subject [197,201]. The patients suffering from DRA had a reduced expression of NKp46, perforin, and granzyme on NK cells [200]. Interestingly, SF NK cells did not express KIRs [198,202].

It remains unclear whether cytokines, hypoxia and other factors of inflammatory joint microenvironment may induce polarization of CD56^bright^ NK cells in RA. Yamin et al. suggest that the CD56^bright^ NK cell subpopulation may preferentially migrate from the peripheral blood to the SF, as evidenced by the increased expression of CCR1 on SF NK cells and a relatively high CCR1 expression on the CD56^dim^ subpopulation compared with that on pNK cells [198]. According to the authors, the CD56^dim^ SF NK subpopulation can differentiate from CD56^bright^ pNK cells. There are discrepancies between investigators in terms of the expression of CXCR3 and CCR5 chemokines on the SF NK cells [198,202]. Half of the CD56^bright^ NK cells from DRA and NDRA patients expressed low Fc affinity CD16 receptors. Interestingly, this phenotype is characteristic for the development and maturation of NK cells [198].

Hypothetically, significantly elevated CD56^bright^ NK cell levels may increase pathological angiogenesis and arthritis in RA patients via proangiogenic cytokine production. To our knowledge, no studies showing proangiogenic properties of CD56^bright^ NK cells in RA have been published. However, NK cells from RA patients have been described to release increased levels of proinflammatory TNF and IFN-ɣ [197,203]. As mentioned above, Lee et al. showed an indirect effect of IFN-ɣ-producing NK cells on angiogenesis in a mouse corneal angiogenesis model, where IFN-ɣ-stimulated macrophages produced VEGF and induced angiogenesis [194].

### 5.4. Ischemic Cardiac Diseases and Stroke

All of the pathologies we described so far (e.g., cancer, RA, endometriosis, and AMD) are associated with abnormal, excessive, and harmful angiogenesis. However, in the course of tissue repair, angiogenesis represents an indispensable phenomenon. NK cells regulate the early inflammatory phase of wound healing and play a role in the subsequent events, such as re-epithelialization, angiogenesis, granulation tissue formation, and remodeling [204]. Even though the putative role of NK cells in wound healing angiogenesis is mainly speculative, it appears possible due to the proangiogenic potential of NK cells and the hypoxic conditions often present in damaged tissues. Also in this case, IFN-ɣ produced by NK cells stimulates a functional differentiation of monocytes/macrophages, which release the proangiogenic factors that are key players in wound-associated angiogenesis [204].

In ischemic cardiac diseases, angiogenesis participates in the healing process. Recent studies revealed a beneficial role of NK cells in cardiac remodeling and heart failure, invoking a growing interest in the therapeutic application of these cells in acute coronary syndrome (ACS) [205,206]. NK cells have been proven not only to prevent cardiac fibrosis in the proliferative phase after cardiac injury but also to promote vessel remodeling [206,207]. In a mouse model of myocardial infarction Bouchentouf et al. demonstrated that an injection of IL-2-stimulated NK cells increases neovascularization and angiogenesis following acute cardiac ischemia [208,209]. Cardiac repair was promoted via α4b7 integrin and killer cell lectin-like receptor 1 (KLRG1). Activated NK cells interacted with VCAM-1 on cardiac endothelial cells (α4b7 integrin/VCAM-1) and disrupted KLRG1 binding to N-cadherin. This resulted in the relocation of β-catenins to the nucleus, abolished cell–cell contact inhibition, and led to the proliferation of endothelial cells and formation of new vessels. Additionally, NK cells appear to play a regulatory role in arteriogenesis in patients suffering from arterial obstructive disease [210]. Experiments on mice with hindlimb ischemia demonstrated that collateral artery development was impaired in the individuals depleted for NK cells (via antibodies) and in NK cell-deficient transgenic mice. The reduction of arteriogenesis was even more profound in mice lacking both NK cells and CD4^+^ lymphocytes.

Another disease which, together with cancer and cardiac pathologies, is a leading cause of disability and mortality, is ischemic stroke (IS). Inflammation-mediated angiogenesis plays a vital role in a long-term recovery of stroke survivors [211]. As a result of the disrupted balance of NK cells in the peripheral blood and peripheral organs in IS patients and in animal models of IS, these cells infiltrate the damaged brain. They become an important bridge participating in a complex crosstalk between the immune and nervous system and play a significant role in post-stroke immunodepression, inflammation, and infections [212]. Individuals’ susceptibility to IS may be increased by NK cells, since the IFN-γ released by activated NK cells contributes to endothelial and vascular function disturbances induced by angiotensin II [213]. Animal studies on rats revealed that in cerebral small vessel disease (cSVD), which is one of IS complications, there are abundant NK cells that infiltrate microvessels [214]. This leads to cerebrovascular inflammation and hypertension-associated cognitive decline. On the other hand, it is tempting to speculate that NK cells may be involved in angiogenesis stimulation in the tissues damaged by IS. Hypoxic conditions in the ischemic brain may be a strong factor reshaping the activity and stimulating the proangiogenic capabilities of NK cells. However, this hypothesis needs meticulous studies on different NK cell populations infiltrating the tissues affected by IS. 

## 6. Concluding Remarks

The proportion of low cytotoxic CD56^bright^ NK cells is significantly increased in various cancer types and other ADDs. This subpopulation of NK cells has proven proangiogenic abilities in solid tumors and decidua. In the TME and UME, hypoxic conditions and a local cocktail of factors, such as TGF-β, GdA, galectins, as well as cell-to-cell interactions determine the loss of cytotoxic and acquisition of proangiogenic properties by NK cells. Excessive amounts of these factors (e.g., TGF-β, GdA) are also present in other ADDs (e.g., RA and endometriosis) and may participate in the conversion of NK cells, which aggravates angiogenesis disorders. Thus, it is plausible that the phenomenon of acquiring proangiogenic and losing cytotoxic properties by NK cells is not limited to cancer but is a common feature of ADDs. There is a need for extensive research concerning proangiogenic properties of NK cells in different ADDs in order to deepen our understanding of the pathogenesis of these diseases and contribute to more effective therapies. 

## Figures and Tables

**Table 1:** Comparison of the dominant peripheral blood NK (pNK) cell subset, decidual NK (dNK) cells, decidual-like tumor-infiltrating and tumor-associated NK (TINK, TANK, respectively) cells, in terms of the phenotype, angiogenic activity, and cytotoxicity.

NK Cell Subset	Phenotype	Proangiogenic Factors	Cytotoxicity
pNK cells	CD56^dim^CD16^bright^CD94^+^CD9^−^ CD49^−^ [9]	−	ADCC↑, perforin↑, granzymes↑, KIR↑, NKG2D↑, NCR↑ [78]
dNK cells	CD56^bright^CD16^−^KIR^+^CD9^+^CD49^+^ [13,68]	HIF1↑, VEGF↑, IL-8↑, angiogenin↑, CXCL12↑, IFNɣ↑, MMP2↑, MMP9↑, TGFβ↑, TNF↑ [9,13,55,58]	degranulation capacity↓ NKp30↓, NKp44↓, NKp80↓, NKG2D↓ [59,79]
Decidual-like TINK cells non-small cell lung cancer (NSCLC) [8], renal cell carcinoma (RCC) [12], breast cancer [11,80], melanoma [11] colorectal cancer (CRC) [10,81] lung adenocarcinoma (pleural effusion) [82]	CD56^bright^CD16^dim/−^ CD56+CD16^−^CD9^+^CD49^+^ CD56^bright^CD16^−^CD49a^+^CD69^+^CD57^low^	VEGFA↑, VEGFB↑, PlGF↑, HIF1↑, Ang2↑, IL-6↑, CCl3↑, CXCL1↑, CCR7↑, CD146R↑ IL-8↑ [8,10,12]	ADCC↓, perforin↓, NKp30↓, NKp44↓, NKp46↓, DNAM-1↓, NKG2A↑, NKG2D↓ CD107a↓ [10,54,81,82,83]
Decidual-like TANK cells prostate cancer [84] colorectal cancer [10]	CD56^bright^CD9^+^CD49a^+^CXCR4^+^ CD56^+^CD16^−^CD9^+^CD49^+^	VEGF↑, angiogenin↑, MMP9↑, TIMP2↑, IL-8, ICAM-1↑, VCAM-1↑ [10,84]	degranulation capacity↓, NKG2D↓ [10,84]

ADCC: antibody-dependent cell mediated cytotoxicity; NCR: natural cytotoxicity receptor; VEGF: vascular endothelial growth factor; PlGF: placenta growth factor; Ang-2: angiopoietin 2; MMP: matrix metalloproteinase; TIMP: tissue inhibitor of metalloproteinase; ICAM-1: intercellular adhesion molecule 1; VCAM-1: vascular cell adhesion molecule 1.

## Data Availability

All data is included in the manuscript.
